# Penetrating Cations Enhance Uncoupling Activity of Anionic Protonophores in Mitochondria

**DOI:** 10.1371/journal.pone.0061902

**Published:** 2013-04-23

**Authors:** Yuri N. Antonenko, Ljudmila S. Khailova, Dmitry A. Knorre, Olga V. Markova, Tatyana I. Rokitskaya, Tatyana M. Ilyasova, Inna I. Severina, Elena A. Kotova, Yulia E. Karavaeva, Anastasia S. Prikhodko, Fedor F. Severin, Vladimir P. Skulachev

**Affiliations:** 1 M.V. Lomonosov Moscow State University, A.N. Belozersky Institute of Physico-Chemical Biology, Moscow, Russia; 2 M.V. Lomonosov Moscow State University, Institute of Mitoengineering, Moscow, Russia; 3 M.V. Lomonosov Moscow State University, Faculty of Bioengineering and Bioinformatics, Moscow, Russia; University of Cambridge, United Kingdom

## Abstract

Protonophorous uncouplers causing a partial decrease in mitochondrial membrane potential are promising candidates for therapeutic applications. Here we showed that hydrophobic penetrating cations specifically targeted to mitochondria in a membrane potential-driven fashion increased proton-translocating activity of the anionic uncouplers 2,4-dinitrophenol (DNP) and carbonylcyanide-*p*-trifluorophenylhydrazone (FCCP). In planar bilayer lipid membranes (BLM) separating two compartments with different pH values, DNP-mediated diffusion potential of H^+^ ions was enhanced in the presence of dodecyltriphenylphosphonium cation (C_12_TPP). The mitochondria-targeted penetrating cations strongly increased DNP- and carbonylcyanide m-chlorophenylhydrazone (CCCP)-mediated steady-state current through BLM when a transmembrane electrical potential difference was applied. Carboxyfluorescein efflux from liposomes initiated by the plastoquinone-containing penetrating cation SkQ1 was inhibited by both DNP and FCCP. Formation of complexes between the cation and CCCP was observed spectophotometrically. In contrast to the less hydrophobic tetraphenylphosphonium cation (TPP), SkQ1 and C_12_TPP promoted the uncoupling action of DNP and FCCP on isolated mitochondria. C_12_TPP and FCCP exhibited a synergistic effect decreasing the membrane potential of mitochondria in yeast cells. The stimulating action of penetrating cations on the protonophore-mediated uncoupling is assumed to be useful for medical applications of low (non-toxic) concentrations of protonophores.

## Introduction

The concept of “mild uncoupling” implies favorable therapeutic action of low concentrations of uncouplers which slightly decrease membrane potential thereby preventing fast production of reactive oxygen species (ROS) in mitochondria [1]–[3]. In fact, low concentrations of the protonophore 2,4-dinitrophenol (DNP) exhibit favorable effects in some pathological laboratory models related to oxidative stress and increase lifespan of yeast [4], *Drosophila* [5] and mice [6].

DNP was used in the early 1900’s as a weight loss-causing drug but was prohibited because of some toxicity and complications resulting in a few deaths (reviewed in [7]). Some efforts have been carried out to the search for uncouplers with a therapeutic window broader than DNP [8]. It has been assumed that toxic action of DNP and its analogues is related to an excessive decrease in mitochondrial membrane potential. However, uncouplers are known to have additional intracellular targets, e.g., plasma membrane potential and ΔpH [9], [10]. It has also been shown that DNP inhibits chloride channel of erythrocytes [11] and blocks glutathione-S-conjugate forming ATP-ase [12]. Comparison of toxic doses for mice with uncoupling concentrations for isolated mitochondria has revealed poor correlation of these parameters for a large set of DNP analogues as well as for some other uncouplers [13]. These findings suggest that the toxic effect of at least some uncouplers *in vivo* could be unrelated to their action on mitochondria. It seems possible that the therapeutic window for uncouplers could be enlarged by agents promoting the uncoupling action of their small concentrations.

It has been shown that protonophorous action of CCCP (carbonyl cyanide m-chlorophenylhydrazone) and FCCP (carbonyl cyanide-p-trifluoromethoxyphenylhydrazone) on lipid vesicles can be enhanced upon the addition of the potassium transporter valinomycin and the penetrating cation tetraphenylphosphonium (TPP) [14]. These effects were explained by formation of complexes between anionic forms of the protonophores and valinomycin-bound K^+^ or TPP cations in lipid membranes [14]–[16]. Addition of the cationic anesthetic procaine to isolated mitochondria led to substantial acceleration of respiration mediated by a CCCP derivative due to the formation of complexes between these compounds [17]. Recently, a new generation of penetrating cations capable of targeted delivery of antioxidants and some other compounds into mitochondria was synthesized and characterized. It was shown that conjugates of penetrating cations and quinones, such as MitoQ and SkQ, protected brain and kidney from ischemia injuries accompanied by generation of ROS [18]–[20]. In the present paper, we demonstrate the enhancing effects of SkQ and related compounds on the protonophorous action of DNP and FCCP in different experimental systems, including model lipid membranes, isolated mitochondria and yeast cells. According to our recent findings [21], SkQ1 (10-(6-plastoquinonyl)decyl triphenylphosphonium) is able to transport organic anions of various structures through lipid bilayer membranes (BLM) and liposomes. In particular, interaction of SkQ with fatty acid anions strongly stimulated the uncoupling effect of the latter on mitochondria [22]. Importantly, fatty acids per se do not exhibit protonophorous action and induce uncoupling via ATP/ADP-antiporter [23]. Below, we show that SkQ and its analogues promote the uncoupling activity of FCCP and DNP much more efficiently than TPP, being operative already at micromolar and even submicromolar concentrations. This suggests that a combined use of uncouplers and mitochondrially-targeted cationic antioxidants may be promising in an attempt to lower effective concentrations of protonophores as therapeutic tools.

## Materials and Methods

### Chemical Substances

**Figure 1:**
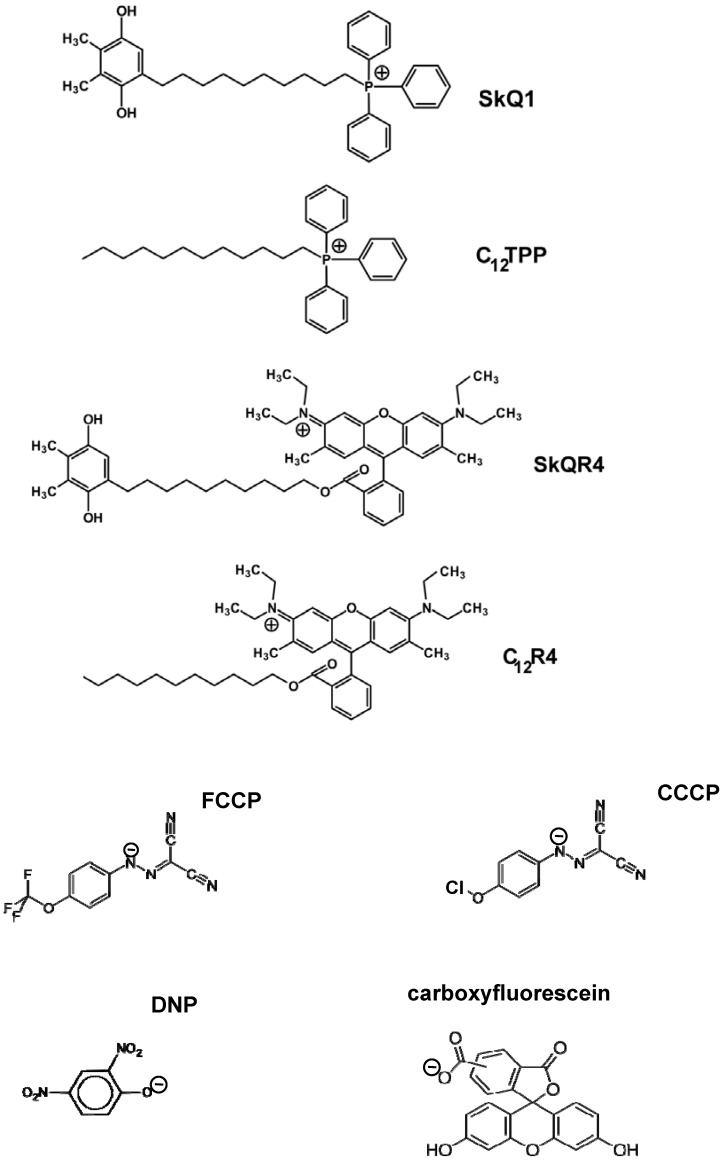
Chemical structures of C_12_TPP, SkQ1, SkQR4, C_12_R4, FCCP, CCCP, DNP, and carboxyfluorescein.

Synthesis of a series of mitochondria-targeted antioxidants composed of substituted 1,4-benzoquinone rings conjugated to hydrophobic triphenylphosphonium (SkQ1) or rhodamine cations (SkQR1) with a decyl linker was described in our group by G.Korshunova and N.Sumbatyan [21], [24]. Structures of SkQ1 and some other compounds used in this work are shown in Fig. 1. FCCP, CCCP, DNP, MOPS, oligomycin, L-malic acid disodium salt, L-glutamic acid potassium salt monohydrate and fatty acid-free BSA were from Sigma-Aldrich; EGTA, safranin O, and potassium phosphate were from Serva; sucrose was purified via twice precipitation from a concentrated solution in ethanol.

### Planar Bilayers

Bilayer lipid membrane (BLM) was formed from 2% decane solution of diphytanoylphosphatidylcholine (DPhPC) on a 0.6-mm aperture in a Teflon septum separating the experimental chamber into two compartments of equal size (volumes, 3 ml). The current measured by a patch-clamp amplifier (OES-2, OPUS, Moscow) was digitized using an NI-DAQmx device (National Instruments, Austin, TX) and analyzed with a personal computer using WinWCP Strathclyde Electrophysiology Software designed by J. Dempster. Electrical potential was measured with two AgCl electrodes placed into the solutions on both sides of the BLM, using a Keithley 6517 amplifier (Cleveland, Ohio, USA). The incubation mixture contained 50 mM Tris-HCl, and 50 mM KCl, pH 7.0.

### Liposome Leakage Assay

Liposomes loaded with 5,6-carboxyfluorescein (CF, Sigma) were prepared by extrusion through a 100-nm filter (Avanti Mini-Extruder) from diphytanoylphosphatidylcholine (Avanti Polar Lipids) in solution containing 100 mM CF titrated with TRIS-base. The unloaded CF was then removed by passage through a Sephadex G-50 coarse column using 100 mM KCl, 10 mM Tris, pH 7.4 as the eluting buffer. To measure the rate of CF efflux, the liposomes were diluted with a buffer containing 100 mM KCl, 10 mM Tris, pH 7.4. The fluorescence at 520 nm (excitation, 490 nm) was monitored with a Panorama Fluorat 02 spectrofluorimeter (Lumex, Russia). At the end of each recording, 0.1% Triton-X100 was added to complete the efflux process.

### Isolation of Rat Liver Mitochondria

Rat liver mitochondria were isolated by differential centrifugation [25] in a medium containing 250 mM sucrose, 10 mM MOPS, 1 mM EGTA, and bovine serum albumin (0.1 mg/ml), pH 7.4. The final washing was performed in the medium of the same composition. Protein concentration was determined using biuret method. Handling of animals and experimental procedures were conducted in accordance with the international guidelines for animal care and use and were approved by the Ethics Committee of A.N. Belozersky Institute of Physico-Chemical Biology at Moscow State University.

### Membrane Potential (ΔΨ) Measurement in Isolated Mitochondria

*ΔΨ* was estimated using the safranin O dye [26]. The difference in the absorbance between at 555 and 523 nm (ΔA) was recorded with an Aminco DW-2000 spectrophotometer in the dual wavelength mode. The following incubation medium was used: 250 mM sucrose, BSA (0.1 mg/ml), a respiratory substrate, 1 mM EGTA, 100 µM potassium phosphate, 10 mM MOPS-KOH (pH 7.4), 10 µM safranin O. The mitochondrial protein content was 0.6–0.9 mg protein/ml, The temperature was 26°C.

### Yeast Strains and Cell Growth Conditions

In our experiments, we used standard laboratory yeast strain W303 (Mat *a* genetic background) and its respiratory incompetent derivative (*petite* strain). The *petite* strain was generated by growing cells on plates supplemented with ethidium bromide [27]. Rich liquid medium contained yeast extract (1%) supplemented with bactopeptone (2%) and 2% glycerol (YPGly medium), or 2% glucose, or 2% glycerol and 0.1% glucose (YP medium) [28]. The cells were typically grown in liquid media to the density of 2×10^6^ cells/ml. For testing the biomass accumulation, the respiratory-incompetent cells were grown for 72 hours to reach the stationary phase in liquid medium supplemented with 0.1% glucose.

### Cell Respiration

Respiration of yeast cells was measured using a standard polarographic technique with a Clark-type oxygen electrode [29]. The incubation medium for yeast cells contained 50 mM KH_2_PO_4_, pH 5.5, and 0.005% glucose.

### Mitochondrial Membrane Potential in Yeast Cells

For estimation of mitochondrial membrane potential, intact yeast cells grown on glycerol-containing rich medium were stained with 2 µM JC-1 [30]. Under our experimental conditions, we did not observe any significant red shifts in fluorescence maxima and, therefore, we assumed that JC-1 did not form J-aggregates. After incubation with JC-1, cells were photographed with an Olympus U-MNIABA3 filter set (excitation, 470–495 nm; split, 505 nm; emission, 510–550 nm) and analyzed with ImageJ software. For each cell, the local intensity maximum was determined. The average intensities of the square areas (0.6 µm^2^) with the center of determined maximum were measured. As a result, an average of JC-1 fluorescence intensity was assigned for individual cells. For each treatment conditions, about 60 cells from a least 2 different experiments were analyzed. The average intensities of local maximum areas of the control (solvent-treated) cells were taken as 100%.

In all figures, the typical results of 4–6 experiments were shown.

## Results

### Diffusion Potential and Electrical Current on Planar Bilayer Lipid Membranes

**Figure 2:**
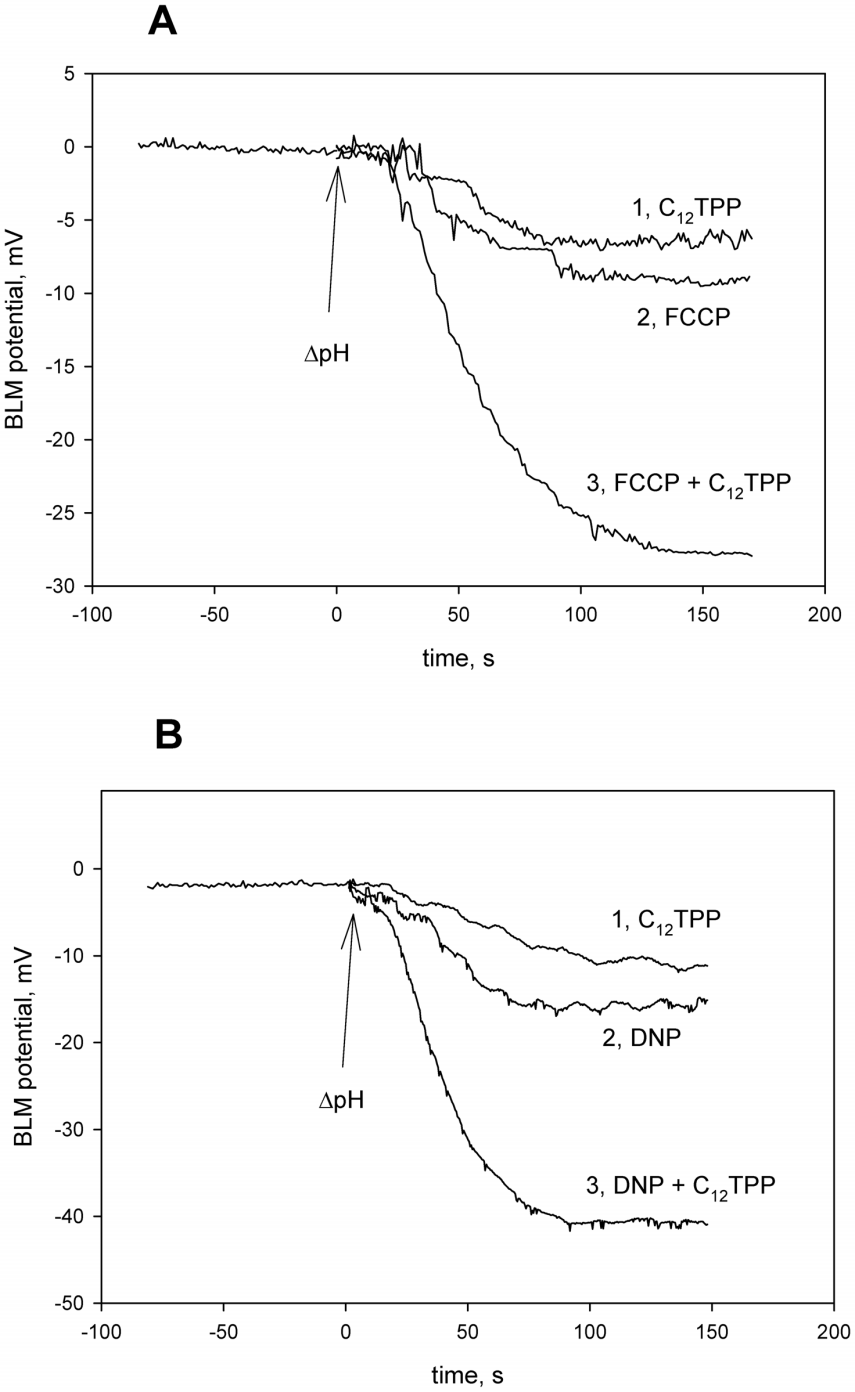
C_12_TPP enhances protonophorous effect of 2 nM FCCP (panel A) and 10 µM DNP (panel B) in bilayer lipid membrane (BLM). Diffusion potential was recorded upon the addition of KOH in one compartment to create ΔpH = 1. Incubation mixture, 10 mM Tris, 10 mM MES, 10 mM KCl, pH 7; C_12_TPP, 0.1 µM. Control, a record without C_12_TPP and uncouplers. Plus sign of the potential in the compartment of high pH.

**Figure 3:**
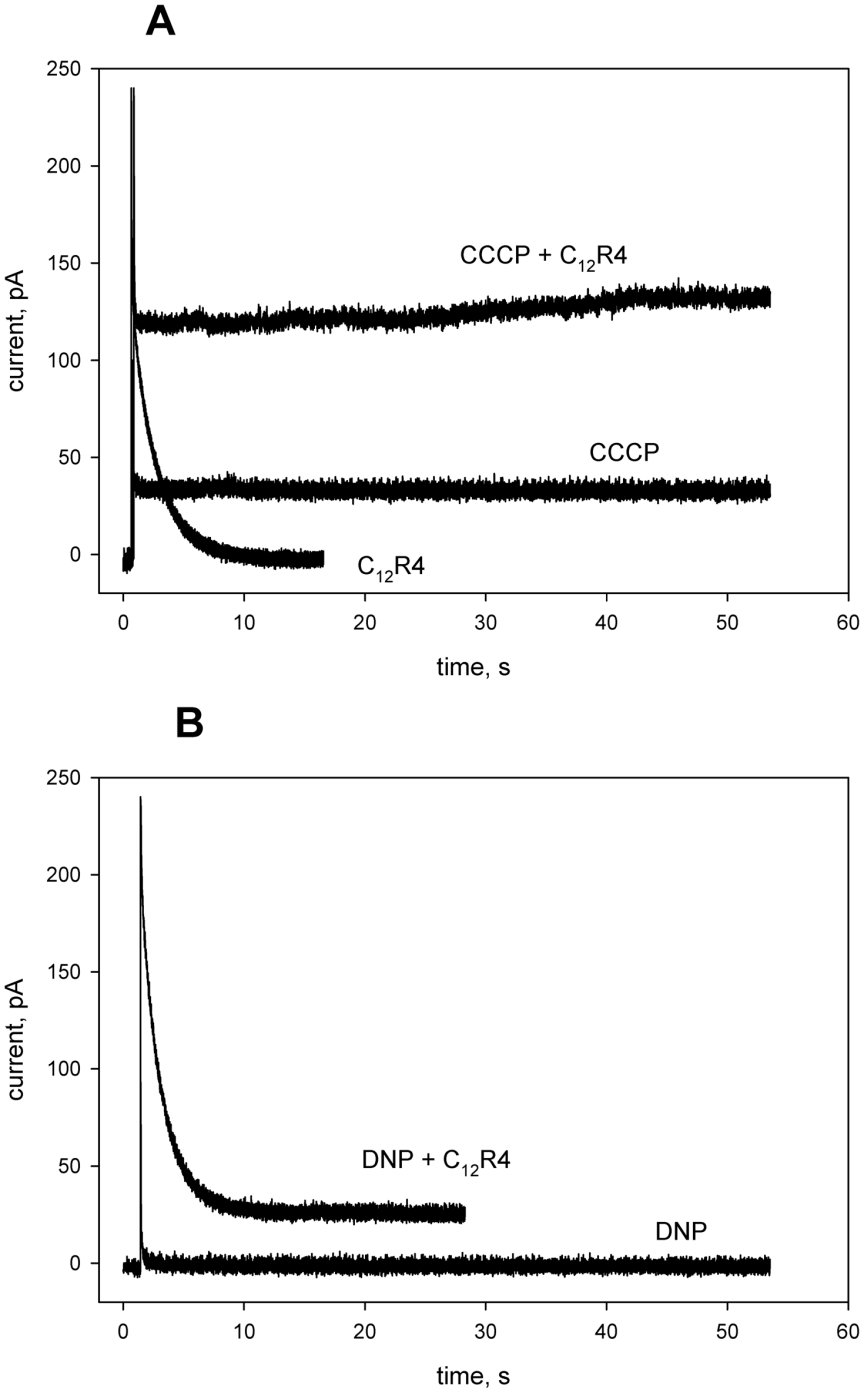
C_12_R4 (rhodamine B dodecyl ester) strongly increases CCCP- (panel A) or DNP-mediated (panel B) electric current through bilayer lipid membrane (BLM). Where indicated, 0.1 µM CCCP or 1 µM DNP were added to both compartments prior to the measurements; incubation mixture, 0.1 M KCl, 10 mM Tris, 10 mM MES, pH 7.0; at t = 1.5 s, 100 mV voltage was applied to the BLM. Concentration of C_12_R4 was 0.1 µM.

To study the effect of the mitochondria-targeted penetrating cations on the uncoupler-mediated proton flux through the planar bilayer lipid membrane (BLM), we measured proton diffusion potential, i.e. an electrical potential difference (ΔΨ) across the BLM, generated as a result of transmembrane H^+^ flux facilitated by a protonophore under the conditions of pH difference between two BLM-separated compartments [31]–[33]. The penetrating cation C_12_TPP substantially increased the value of H^+^ diffusion potential if added in combination with FCCP (Fig. 2A) or DNP (Fig. 2B). In these experiments, protonophores (FCCP or DNP) were added at low concentrations which were far below the level sufficient to generate the theoretical (Nernstian) potential, i.e. 59 mV at ΔpH = 1. However, in the presence of 0.1 µM C_12_TPP, the magnitude of ΔΨ increased and reached approximately 28 mV in the case of FCCP and 42 mV in the case of DNP. The growth of ΔΨ proved that BLM preserved proton selectivity under these conditions. C_12_TPP alone generated only a small diffusion H^+^ potential (Fig. 3A). Importantly, TPP was unable to substitute for C_12_TPP in these experiments (data not shown). Higher concentrations of FCCP or DNP allowed us to observe theoretical (about 60 mV) values of ΔΨ even without hydrophobic cations (not shown).

In further experiments, we applied the current relaxation technique to measure the protonophore-mediated electric current across planar BLM under voltage-clamp conditions. As was shown previously in our group, voltage application to a planar BLM in the presence of SkQ1 or related penetrating cations results in a jump of electric current followed by its relaxation [21], [34]. With rhodamine B dodecyl ester (C_12_R4, Fig. 1), current across the membrane, which was maximal immediately after application of the voltage, also decreased spontaneously with time from an initial to a steady-state level (the current relaxation process). Figure 3 shows current relaxation recordings obtained upon a voltage jump of 100 mV with CCCP (panel A) or DNP (panel B) added symmetrically to the bathing solution in the absence and in the presence of C_12_R4. Note that the steady-state level of electric current with C_12_R4 was negligible if no protonophore (CCCP) was added. The current was higher in the samples with 0.1 µM CCCP. The addition of 0.1 µM C_12_R4 to the CCCP-containing sample led to an increase in the steady-state current from about 40 pA up to 120 pA. An experiment with another protonophore, DNP, showed similar results: the addition of C_12_R4 led to an increase in the steady-state current, and the magnitude of the effect was even higher than with FCCP, namely about 30-fold.

### Spectrophotometrical Evidence for Interactions of a Protonophorous Uncoupler and Penetrating Ions

**Figure 4:**
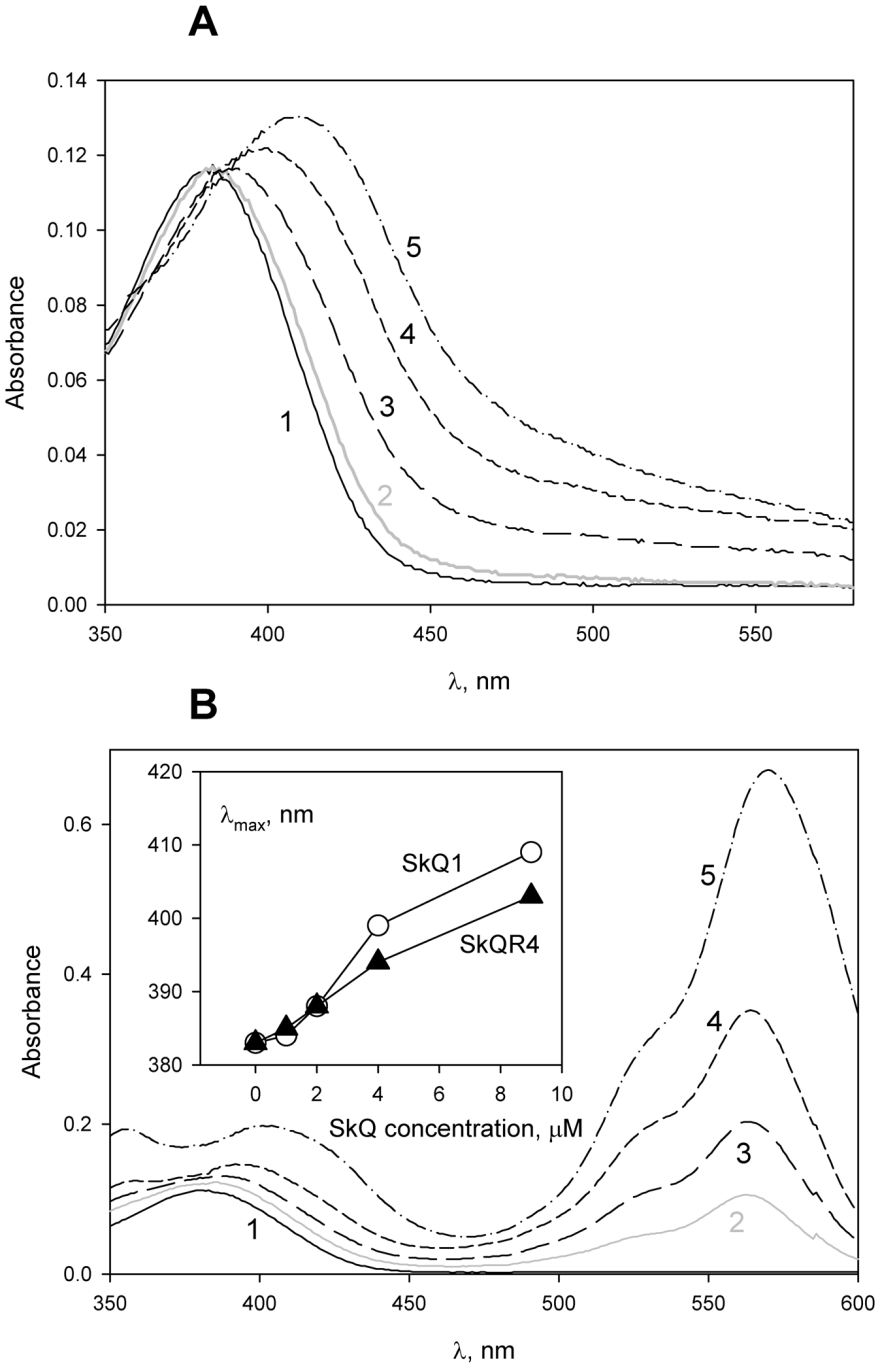
SkQ1 (10-(6-plastoquinonyl)decyl triphenylphosphonium) affects absorption spectra of CCCP in the presence of liposomes. Incubation mixture: 4 µM CCCP, 1 mM Tris, 1 mM MES, pH = 7.4, containing DPhPC (diphytanoylphosphatidylcholine) liposomes (20 µg/ml) in the absence (curve 1) and in the presence of 1 µM, 2 µM, 4 µM and 9 µM SkQ1 (panel A, curves 2–5, respectively) or in the presence of 1 µM, 2 µM, 4 µM and 9 µM SkQR4 (panel B, curves 2–5, respectively). Insert to panel B shows the dependence of λ_max_ on the concentration of the SkQ derivatives.

Data on planar lipid membranes favored the idea of hydrophobic cations being carriers of anionic forms of protonophores. The carrier-type mechanism of transport implies formation of complexes between a substrate (protonophore anion) and a hydrophobic cation. The interaction between CCCP and penetrating cations was studied by measuring CCCP absorption spectra in the presence of SkQ1 (Fig. 4A) and SkQR4 (Fig. 4B) in a suspension of liposomes. The addition of the hydrophobic cations led to a red shift in the absorption maximum (λ_max_) of CCCP, indicating formation of complexes.

### Effect of CCCP and DNP on the SkQ1-induced Carboxyfluorescein Efflux from Liposomes

**Figure 5:**
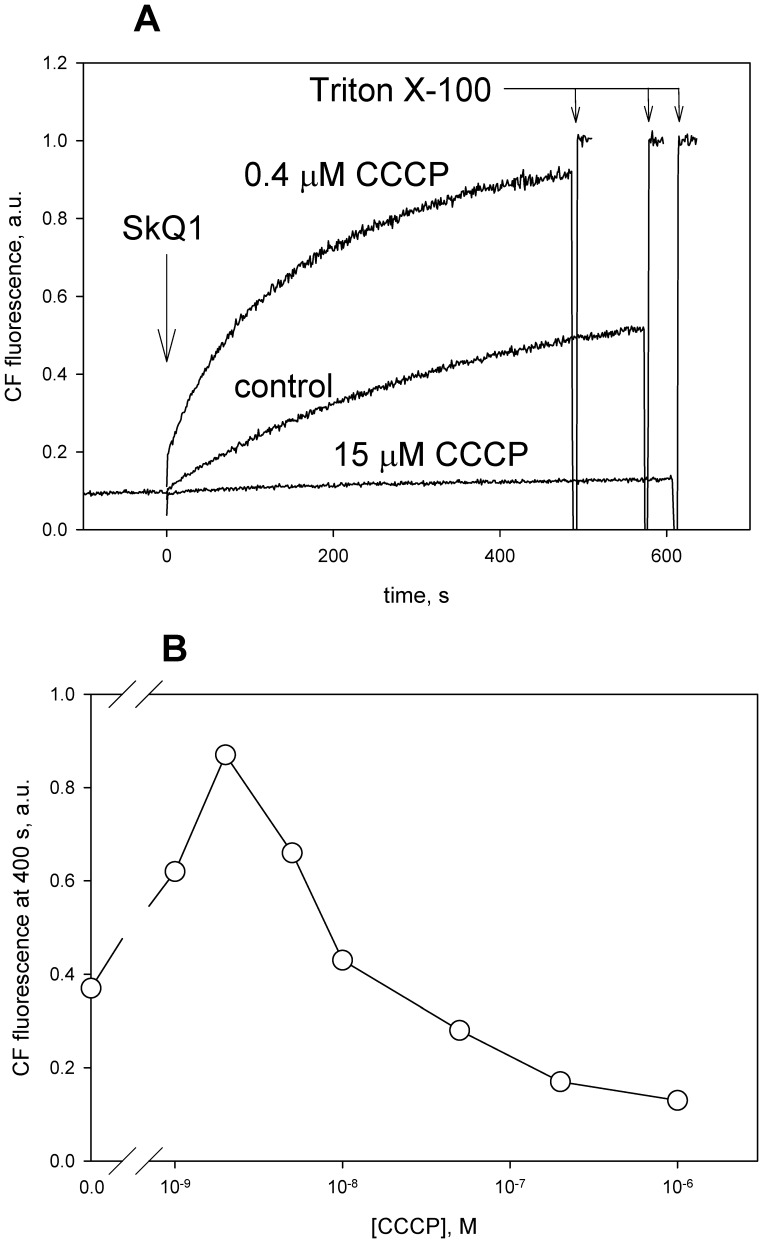
CCCP increases the SkQ-induced efflux of carboxyfluorescein from liposomes. Carboxyfluorescein (CF) efflux from DPhPC (diphytanoylphosphatidylcholine) liposomes (50 µg/ml), induced by 2.5 µM SkQ1 (panel A) or by 0.5 µM SkQR1 (panel B) was measured with or without CCCP. The efflux was accompanied by an increase in CF fluorescence due to dilution and a relief of CF self-quenching. In panel B, the CF efflux was measured 400 s after the addition of SkQR1. Incubation mixture, 10 mM Tris, 10 mM MES, 100 mM KCl, pH 7.

It was previously shown in our group that SkQ1 and other penetrating cations initiated efflux of carboxyfluorescein (CF) anions from liposomes via the carrier-type mechanism [21]. Importantly, the ability to induce the efflux of CF strongly depended on the number of carbon atoms in the alkyl chain (*n*) of alkyltriphenylphosphonium being maximal at *n* about 10–12 [21]. The process was inhibited by fatty acids which competed with CF for the formation of a complex with SkQ1. It could be expected that the anionic protonophores would inhibit the CF efflux as well. Fig. 5 shows the effect of CCCP on SkQ1-induced CF efflux from phosphatidylcholine liposomes loaded with CF at a self-quenching concentration. Efflux of CF from liposomes entailed dilution of CF which increased CF fluorescence. As expected, high concentrations of CCCP blocked the SkQ1-induced CF efflux. However, low CCCP concentrations accelerated the process (top curve in Fig. 5A). Two phases in the concentration dependence were also observed for another penetrating cation, SkQR1 (Fig. 5B) and were also found with DNP (data not shown). The accelerating effect of low concentrations of CCCP or DNP might be associated with the electrogenic nature of CF efflux which should result in the formation of a diffusion potential on membranes of liposomes. Apparently, addition of protonophores decreases this ΔΨ, thus stimulating the CF efflux. As to the inhibitory effect of high concentrations of the protonophores, it could result from formation of their complexes with the penetrating cations.

### Effect of Penetrating Cations on DNP- and FCCP-mediated Uncoupling of Isolated Rat Liver Mitochondria

**Figure 6:**
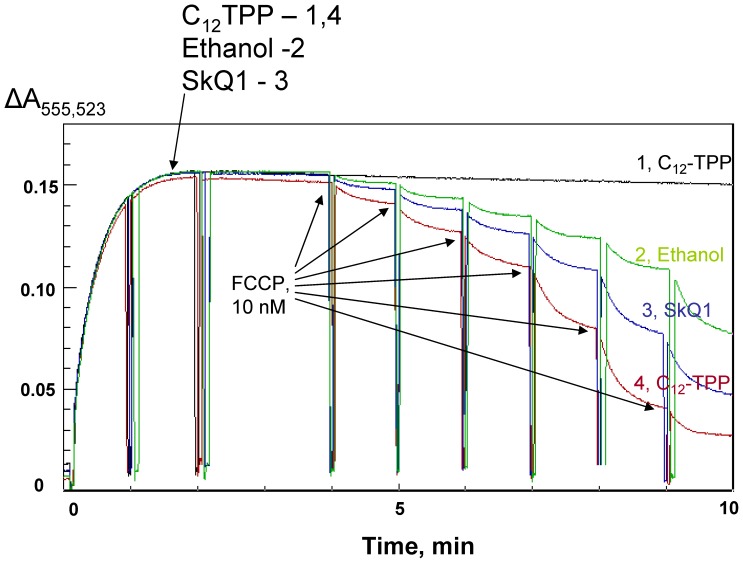
SkQ1 and C_12_TPP stimulate the uncoupling action of FCCP (carbonyl cyanide-p-trifluoromethoxyphenylhydrazone) in rat liver mitochondria. Mitochondrial potential was assayed by measuring the absorbance of safranin O. 10 nM FCCP was added at 4, 5, 6, 7, 8, and 9 min (curves 2–4). 0.2% ethanol or ethanol solutions leading to 2 µM C_12_TPP (curves 1 and 4) or 2 µM SkQ1 (curve 3) were added at 2 min. Oligomycin (1 µg/ml) was added at 1 min. For other conditions, see Materials and methods.

**Figure 7:**
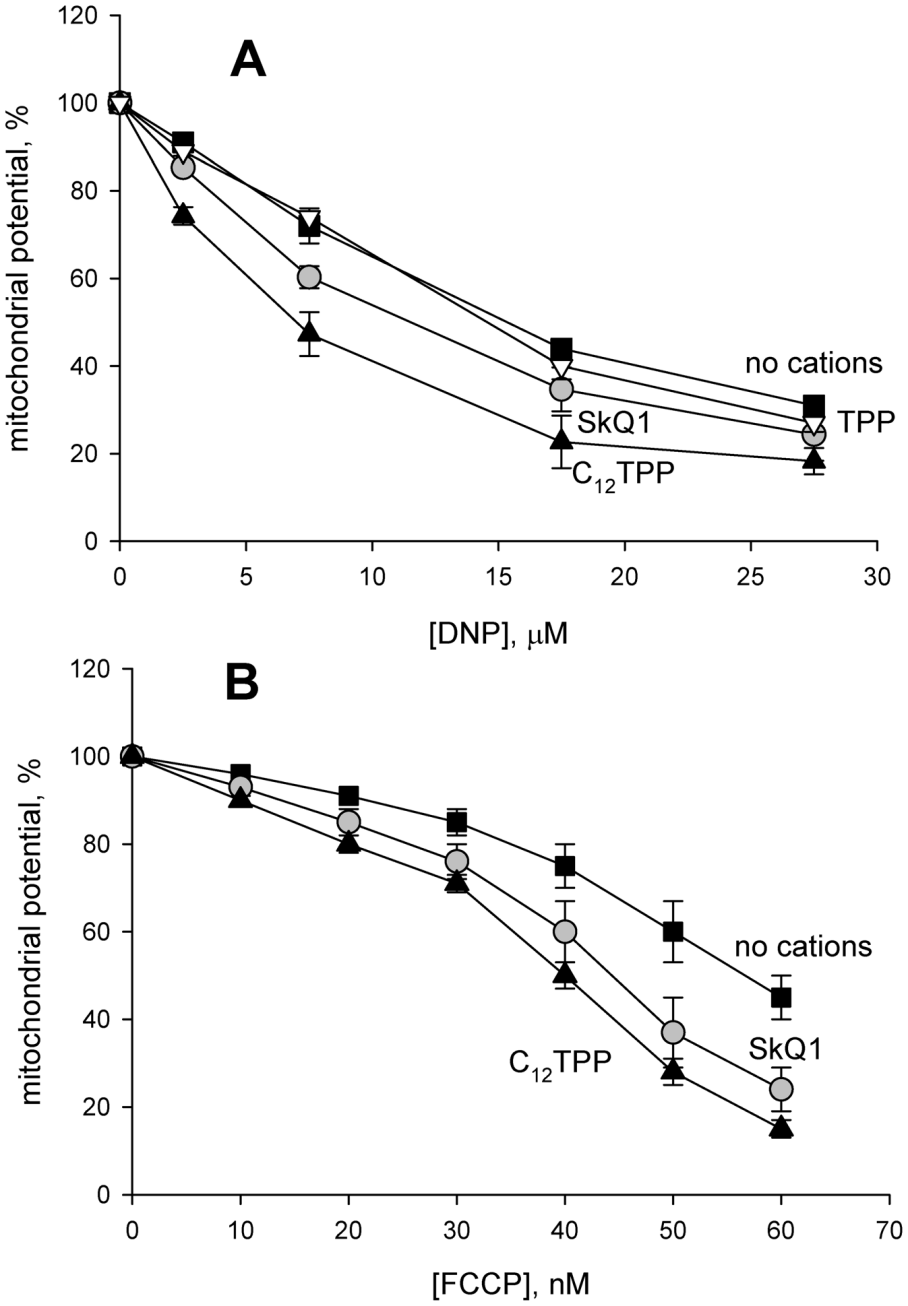
Comparison of SkQ1 and C_12_TPP effects on the uncoupling activity of DNP and FCCP. Effects of 2 µM SkQ1, 2 µM C_12_TPP or 200 µM TPP on the dependence of mitochondrial membrane potential on the concentration of DNP (panel A) or FCCP (panel B). The experiments were conducted in a way shown in Fig. 6. Shown are Mean±S.E. of 4–6 experiments.

Transport of electrons along the mitochondrial respiratory chain is accompanied by the formation of electrochemical gradient of hydrogen ions (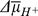)at the inner mitochondrial membrane. The electrical part of 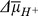, i.e. ΔΨ, can be measured in isolated mitochondria by changes in the absorbance of the cationic dye safranin O [26]. Fig. 6 shows the results of ΔΨ measurements on rat liver mitochondria upon the addition of dodecyl tetraphenylphosphonium cation (C_12_-TPP, curve 1), FCCP (curve 2) or FCCP together with SkQ1 (curve 3) or C_12_-TPP (curve 4). One can see that C_12_-TPP did not decrease ΔΨ under the experimental conditions but substantially increased the FCCP-induced ΔΨ lowering. Fig. 7A and 7B show the dose dependence of the ΔΨ decrease by DNP and FCCP. In contrast to C_12_-TPP, the less hydrophobic cation TPP had no effect even at a concentration of 200 µM (Fig. 7A).

### Effect of Penetrating Cations on FCCP- and DNP-mediated Uncoupling of Mitochondria in Intact Yeast Cells

To test whether the penetrating cations enhanced the action of uncouplers at a cellular level, we studied the effects of C_12_TPP, FCCP and DNP on intact yeast cells. Unlike isolated mitochondria and artificial planar bilayer membranes or liposomes, intact eukaryotic cells have several membraneous compartments differing in values of transmembrane proton gradient. Therefore, the effects of uncouplers on intact cells could be rather complicated. To address this question, we first studied the combined action of the uncouplers and penetrating cations on the ability of *Saccharomyces cerevisiae* cells to accumulate the hydrophobic cationic fluorescent dye JC-1, that is widely used to estimate ΔΨ on the mitochondrial inner membrane in living cells [30].

**Figure 8:**
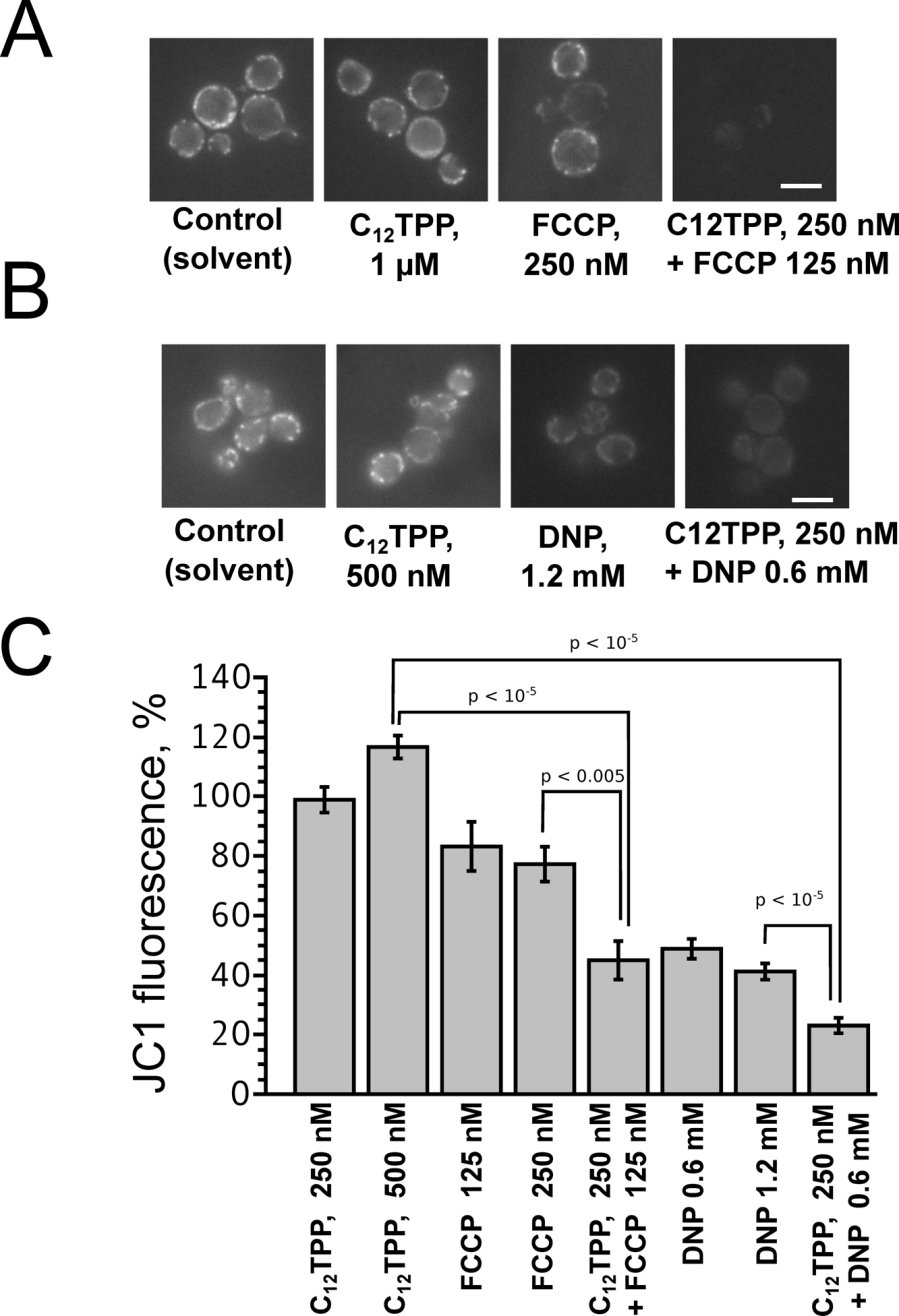
C_12_TPP augments the FCCP- and DNP-induced decrease of mitochondrial membrane potential (ΔΨ) in intact yeast cells. Mitochondrial membrane potential in yeast was estimated by staining cells with the ΔΨ probe JC-1. *S.cerevisiae* cells were incubated with C_12_TPP and/or FCCP (A), C_12_TPP and/or DNP (B) and then loaded with 2 µM JC-1. (C) Levels of JC-1 fluorescence in yeast cells (see Materials and methods). Data are presented as averages with standard errors. Samples were compared by Wilcoxon signed ranked unpaired test. Results of at least three experiments performed on separate days (number of cells, from 30 to 170). Bar, 5 µm.

C_12_TPP added together with the anionic uncouplers FCCP (Fig. 8 A,C) or DNP (Fig. 8 B,C) significantly enhanced the uncoupling effect of these protonophores in yeast mitochondria, as judged by the accumulation of JC-1. Importantly, C_12_TPP added alone in submicromolar concentrations did not affect the ability of yeast cells to accumulate JC-1 (Fig. 8).

**Figure 9:**
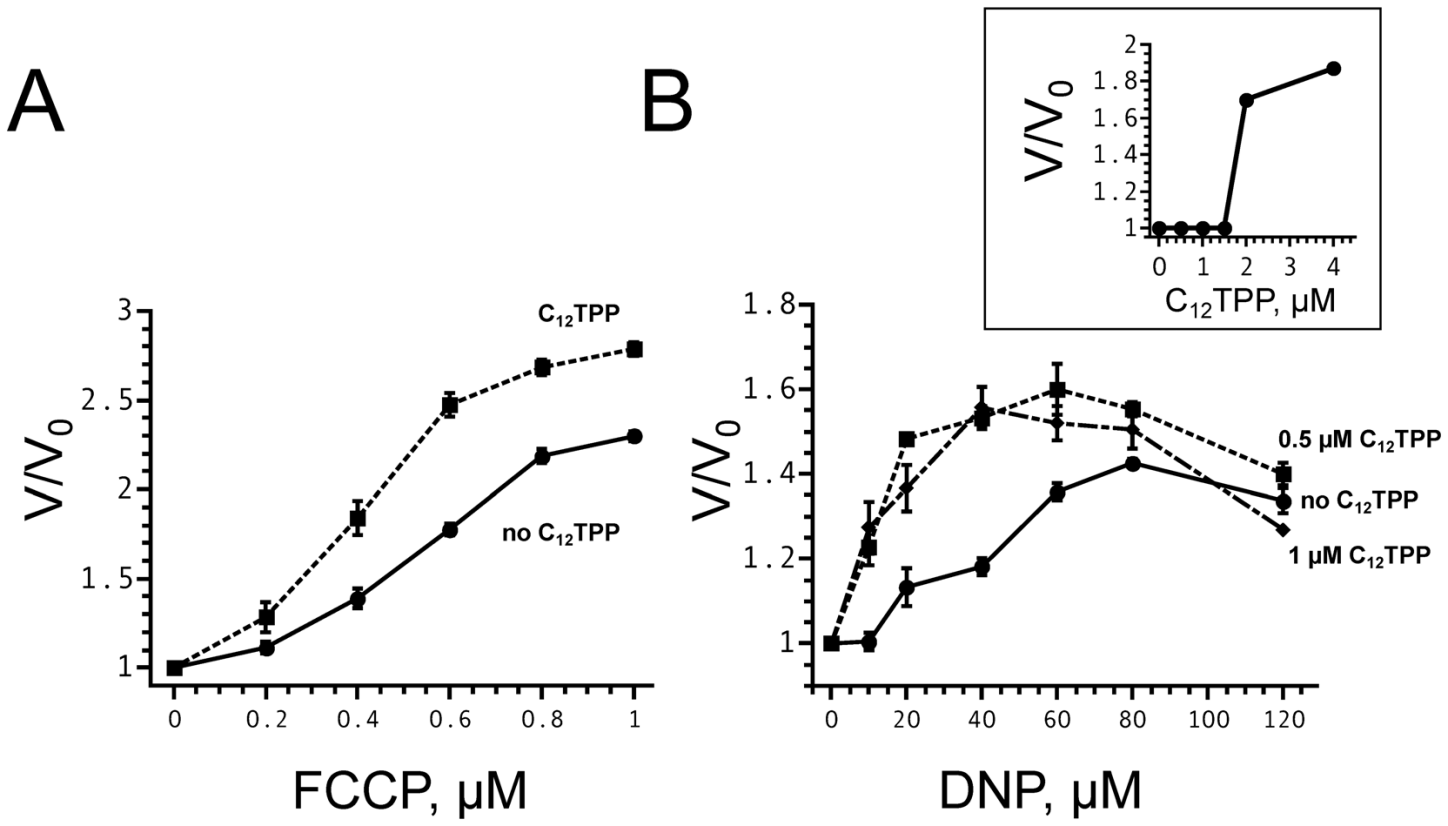
C_12_TPP enlarges the FCCP- and DNP-induced increase in respiration rates of intact yeast cells. The ordinate represents the ratio of the oxygen consumption rates after (V) and before (V_0_) the addition of an uncoupler. (A) FCCP-mediated stimulation of yeast respiration in the absence (solid line) and in the presence (dotted line) of 1 µM C_12_TPP. (B) DNP stimulation of yeast respiration in the absence (solid line) and in the presence of 0.5 µM (dotted line) or 1 µM (dot and dash line) C_12_TPP. In the absence of the anionic uncoupler, C_12_TPP did not increase the rate of oxygen consumption at concentrations below 2 µM (insert). Shown are Mean±S.E. of 4 experiments.

Figure 9A shows the dependence of the yeast cell respiration rate on the concentration of FCCP in the absence (solid line) and in the presence (dotted line) of 1 µM C_12_TPP. It is seen that the combination of C_12_TPP and FCCP stimulated respiration stronger than FCCP alone. Under these conditions, C_12_TPP per se did not produce any effect up to concentrations of 2 µM (insert), in agreement with earlier data [8]. C_12_TPP activated the DNP-mediated respiration too (Figure 9B).

## Discussion

Summarizing the above presented data, one can conclude that C_12_-TPP, SkQ1 and other hydrophobic penetrating cations are able to promote the protonophorous action of anionic uncouplers on model lipid membranes (Figs.2, 3) and certain natural (mitochondrial) membranes (Figs.6, 7, 8, and 9). The planar bilayer experiments revealed a stimulating effect of the penetrating cations both on the uncoupler-assisted transmembrane proton flux manifesting itself in augmenting the H^+^ diffusion potentials on BLM (Fig. 2) and on the steady-state current in voltage-jump relaxation experiments (Fig. 3) reflecting an increase in the transmembrane flux of charged forms of an uncoupler [33]. Spectral measurements (Fig. 4) together with liposome leakage experiments (Fig. 5) allowed us to shed light on the mechanism of the uncoupler activation, most likely proceeding via formation of complexes between the penetrating cations and anionic forms of the uncouplers. High hydrophobicity of cation and anion seems to be required for formation of such complexes because electrostatic interactions are much stronger in hydrophobic membrane core than in water solutions. In line with this assumption, the cation of tetraphenylphosphonium (TPP), being rather hydrophilic, was of no effect in our systems in contrast to C_12_TPP (Fig. 7A). As the penetrating cations are known to accumulate electrophoretically in mitochondria [35]–[38], their promoting effect on the action of DNP or FCCP should be more pronounced at low uncoupler concentrations which decrease ΔΨ to a small extent. Importantly, our planar bilayer data showed that the influence of penetrating cations was not associated with their possible detergent effects because they (i) did not induce fluctuations of the electric current (Fig. 3), and (ii) did not reduce proton selectivity of the membrane (Fig. 2).

It was previously found that penetrating cations stimulated uncoupling action of endogeneous free fatty acids present in mitochondria [22]. In this paper, we used comparatively low concentrations of SkQ1, C_12_TPP and other cations which did not affect the mitochondrial ΔΨ (Figs.6) or decreased it only slightly (Figs.7). Importantly, the combined action of SkQ1 (or C_12_TPP) and DNP (or FCCP) was stronger, as compared to the sum of their separate effects (Figs.6, 8, 9). This observation supported our conclusion that direct interaction of the studied cations and artificial uncouplers in the membrane caused their synergistic action.

Although C_12_TPP increased the uncoupling action of FCCP and DNP on *S. cerevisiae* cells (Fig. 8, 9), it did not enhance inhibiting effects of anionic uncouplers on biomass accumulation (see Supplemental information, Fig. S1 and S2). Apparently, the stationary biomass level was controlled primarily by the efficiency of ATP production. Therefore, it can be concluded that the combination of C_12_TPP and anionic uncouplers, while decreasing ΔΨ in tightly coupled fraction of mitochondria, still did not significantly inhibit the rate of ATP synthesis. In other words, DNP and FCCP, at small concentrations, being supplemented with C_12_TPP, become more efficient but still remain rather mild uncouplers. Hence, the promoting action of penetrating cations on the uncoupling effect of anionic protonophores seems promising for the development of drugs against obesity. Known uncouplers display relatively narrow windows between favorable and toxic concentrations which makes their medical use problematic [8]. It is generally accepted that the major cause of such toxicity is inhibition of oxidative phosphorylation. On the other hand, DNP and some other uncouplers are known to decrease the electrical potential on plasma membranes of erythrocytes [9], [10]. These and other data suggest that toxicity of uncouplers, apart from their action on oxidative phosphorylation, is at least partially unrelated to mitochondria. Our data on the inhibition of the yeast cell growth (Figs. S1 and S2) support the idea of non-mitochondrial toxic action of DNP and FCCP, which is in line with results of Ilivichy and Casida [13] showing poor correlation between the effect of DNP and its derivatives on the respiration of isolated mitochondria and the toxic action on mice. In particular, LD50 for toxicity of DNP and 6-cyclohexyl-DNP differed 1.5-fold, while uncoupling concentrations differed more than 30-fold [13]. It is essential that penetrating cations, being mitochondria-targeted compounds, should specifically stimulate mitochondria-linked activity of uncouplers rather than any other effects. In any case, there is a chance that the use of DNP in combination with hydrophobic penetrating cations as an obesity-lowering drug could be effective at substantially smaller (non-toxic) DNP doses. This idea seems to be even more attractive if one takes into account the data on several beneficial effects of SkQ or MitoQ used separately in a number of animal models of various diseases [18], [19].

## Supporting Information

Figure S1 **FCCP and C_12_TPP do not display synergistic inhibition of the increase in biomass of** ***S. cerevisiae*** **.** Yeast cells were incubated in YPGly medium (see “Materials and methods”) supplemented with C_12_TPP and FCCP separately or with equimolar mixture of these compounds. For “C_12_TPP+FCCP” curve, the sum of the concentrations is shown, i.e. the data point “2 µM” corresponds to the growth medium supplemented with 1 µM of each compound. Incubation time was 5 hours. After incubation, the optical density (OD) was measured (λ = 550 nm). The OD of mock treated probe was set as 100%. Figure S1 shows that C_12_TPP did not enhance the inhibitory effect of FCCP on yeast growth, if the cells were grown on nonfermentable carbon source (glycerol). This result suggests that FCCP inhibited the growth not via arrest of oxidative phosphorylation but rather due to some other activity, for instance due to changing the proton potential on the plasma or vacuolar membranes.
(TIFF)

Figure S2 **C_12_TPP did not affect the ability of neither FCCP (A) nor DNP (B) to decrease optical density of yeast respiratory-incompetent cells grown up to the stationary phase.** The cells were incubated in YP medium for 72 hours. Respiratory incompetent cells were taken to ensure that ethanol (added as solvent of uncouplers and C_12_TPP) was not used by cells as a carbon source. Curves in the left plots represent the separate effects of the tested compounds, the right bar plots indicate the combined effects of each uncoupler supplemented with C_l2_TPP in concentrations shown by the tables below the graphs. The OD of the mock-treated probe was set as 100%. In the experiments shown in Figure S2, we used petite (respiratory incompetent) yeast strain cells which were not able to utilize 0.1% ethanol added to the medium as a mock or as a solvent of uncouplers. Moreover, it is shown that in this system C_12_TPP did not increase the efficiency of the inhibition of cell growth by the anionic uncouplers. One may speculate that in this case inhibition of cell growth by uncouplers was also a result of their action on the plasma or vacuolar membrane rather than on mitochondria. Indeed, partial inactivation of Pma1p (plasma membrane H^+^-ATPase) was reported to increase the level of ATP in yeast cells (Holyoak et al., 1996 Appl Environ Microbiol 62: 3158-3164.). This indicates that maintenance of proton gradient on a plasma membrane is highly energy-consuming process and dissipation of this gradient could result in a decrease of biomass production.
(TIFF)
