# Supplementary figures and images for: Penetrating Cations Enhance Uncoupling Activity of Anionic Protonophores in Mitochondria
Source: PLoS One. 2013 Apr 23;8(4):e61902. doi: 10.1371/journal.pone.0061902 (PMC3633956; doi:10.1371/journal.pone.0061902)

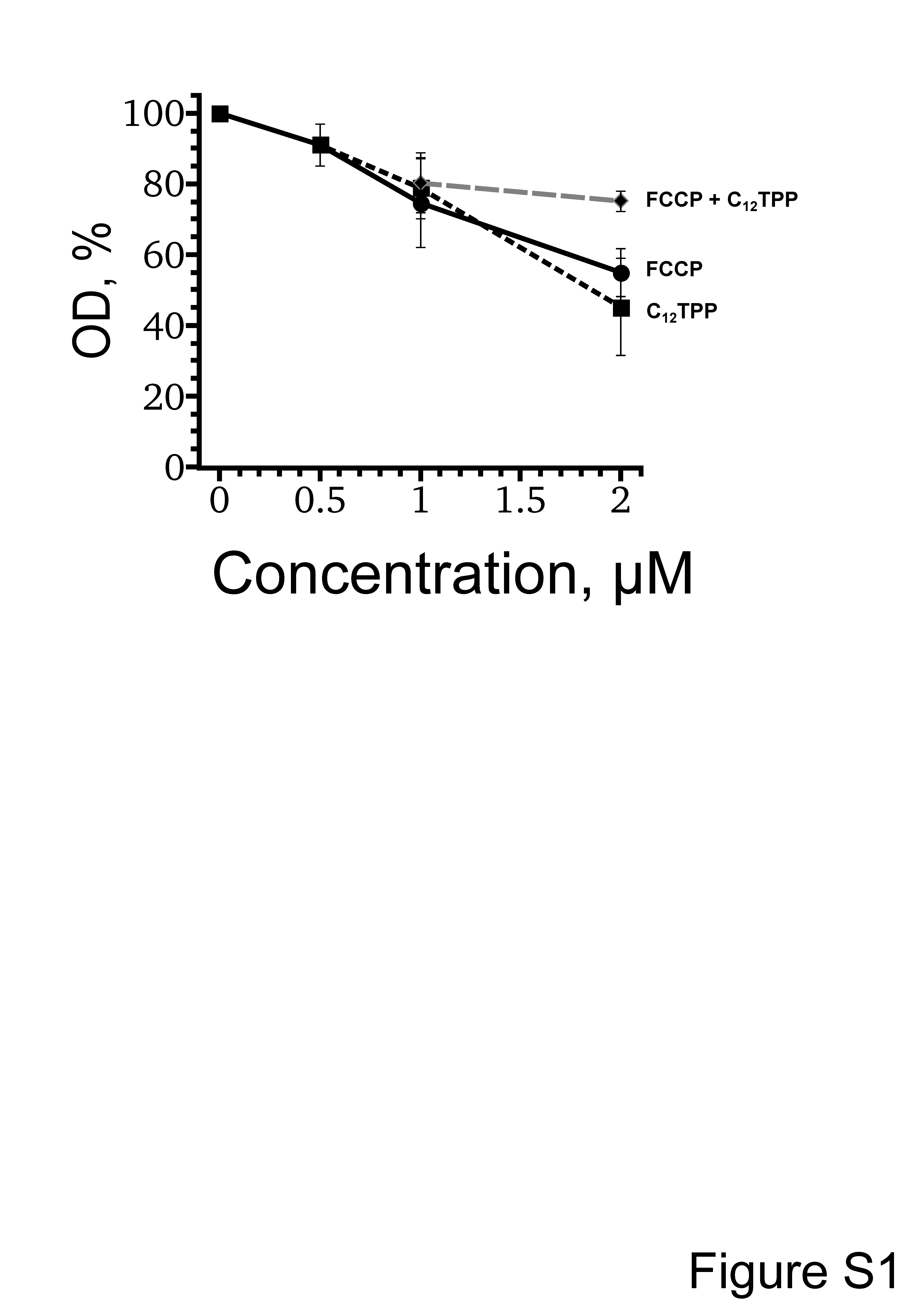

Supplement: Figure S1 — FCCP and C12TPP do not display synergistic inhibition of the increase in biomass of S. cerevisiae . Yeast cells were incubated in YPGly medium (see “Materials and methods”) supplemented with C12TPP and FCCP separately or with equimolar mixture of these compounds. For “C12TPP+FCCP” curve, the sum of the concentrations is shown, i.e. the data point “2 µM” corresponds to the growth medium supplemented with 1 µM of each compound. Incubation time was 5 hours. After incubation, the optical density (OD) was measured (λ = 550 nm). The OD of mock treated probe was set as 100%. Figure S1 shows that C12TPP did not enhance the inhibitory effect of FCCP on yeast growth, if the cells were grown on nonfermentable carbon source (glycerol). This result suggests that FCCP inhibited the growth not via arrest of oxidative phosphorylation but rather due to some other activity, for instance due to changing the proton potential on the plasma or vacuolar membranes. (TIFF) [file pone.0061902.s001.tiff]

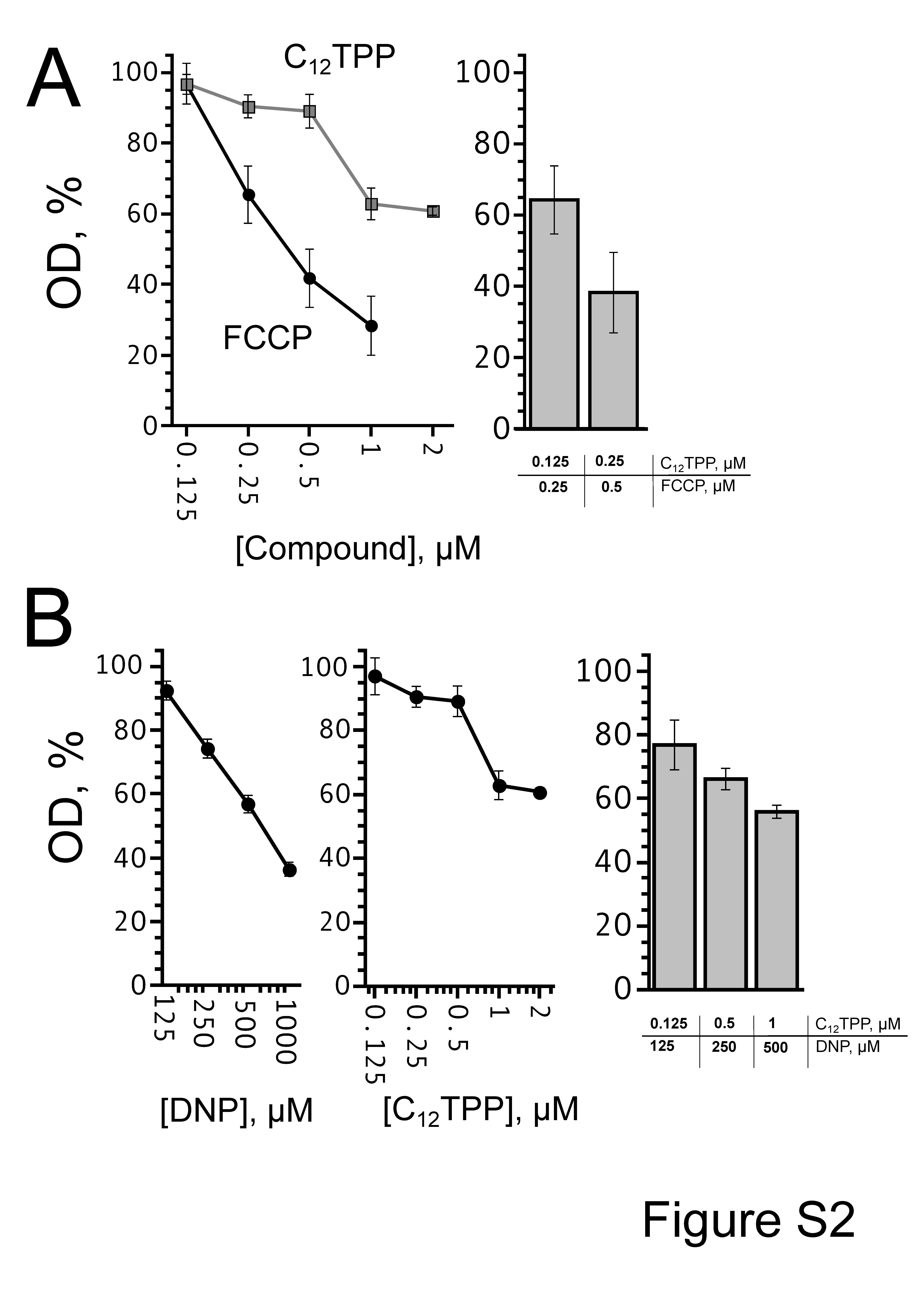

Supplement: Figure S2 — C12TPP did not affect the ability of neither FCCP (A) nor DNP (B) to decrease optical density of yeast respiratory-incompetent cells grown up to the stationary phase. The cells were incubated in YP medium for 72 hours. Respiratory incompetent cells were taken to ensure that ethanol (added as solvent of uncouplers and C12TPP) was not used by cells as a carbon source. Curves in the left plots represent the separate effects of the tested compounds, the right bar plots indicate the combined effects of each uncoupler supplemented with Cl2TPP in concentrations shown by the tables below the graphs. The OD of the mock-treated probe was set as 100%. In the experiments shown in Figure S2, we used petite (respiratory incompetent) yeast strain cells which were not able to utilize 0.1% ethanol added to the medium as a mock or as a solvent of uncouplers. Moreover, it is shown that in this system C12TPP did not increase the efficiency of the inhibition of cell growth by the anionic uncouplers. One may speculate that in this case inhibition of cell growth by uncouplers was also a result of their action on the plasma or vacuolar membrane rather than on mitochondria. Indeed, partial inactivation of Pma1p (plasma membrane H+-ATPase) was reported to increase the level of ATP in yeast cells (Holyoak et al., 1996 Appl Environ Microbiol 62: 3158-3164.). This indicates that maintenance of proton gradient on a plasma membrane is highly energy-consuming process and dissipation of this gradient could result in a decrease of biomass production. (TIFF) [file pone.0061902.s002.tiff]
